# Patterns and Predictors of Firearm-related Spinal Cord Injuries in Adult Trauma Patients

**DOI:** 10.5811/westjem.2020.9.48202

**Published:** 2021-02-15

**Authors:** Dina Mahmassani, Rana Bachir, Mazen El Sayed

**Affiliations:** American University of Beirut Medical Center, Department of Emergency Medicine, Beirut, Lebanon

## Abstract

**Introduction:**

Firearm-related spinal cord injuries are commonly missed in the initial assessment as they are often obscured by concomitant injuries and emergent trauma management. These injuries, however, have a significant health and financial impact. The objective of this study was to examine firearm-related spinal cord injuries and identify predictors of presence of such injuries in adult trauma patients.

**Methods:**

This retrospective cohort study examined adult trauma patients (≥16 years) with injuries from firearms included in the 2015 United States National Trauma Data Bank. We performed descriptive and bivariate analyses and compared two groups: patients with no spinal cord injury (SCI) or vertebral column injury (VCI); and patients with SCI and/or VCI. Predictors of SCI and/or VCI in patients with firearm-related injuries were identified using a multivariate logistic regression analysis.

**Results:**

There were 34,898 patients who sustained a firearm-induced injury. SCI and/or VCI were present in 2768 (7.9%) patients. Patients with SCI and/or VCI had more frequently severe injuries, higher Injury Severity Score (ISS), lower mean systolic blood pressure, and lower Glasgow Coma Scale (GCS). The mortality rate was not significantly different between the two groups (14.7%, N = 407 in SCI and/or VCI vs 15.0%, N = 4,811 in no SCI or VCI group). Significant general positive predictors of presence of SCI and/or VCI were as follows: university hospital; assault; public or unspecified location of injury; drug use; air medical transport; and Medicaid coverage. Significant clinical positive predictors included fractures, torso injuries, blood vessel or internal organ injuries, open wounds, mild (13–15) and moderate GCS scores (9 – 12), and ISS ≥ 16.

**Conclusion:**

Firearm-induced SCI and/or VCI injuries have a high burden on affected victims. The identified predictors for the presence of SCI and/or VCI injuries can help with early detection, avoiding management delays, and improving outcomes. Further studies defining the impact of each predictor are needed.

## INTRODUCTION

### Background

Firearm-related injuries continue to have a significant health and financial impact worldwide. In the United States (US), mass shootings are responsible for increasing proportions of total firearm-related homicidal deaths.^1^ In 2017, the rate of nonfatal, firearm-related gunshot injuries was 41.1 per 100,000 injured.^2^ The fatality rate of firearm-related gunshot injuries was 12.2 per 100,000 injuries.^3^ Between the years 2006 and 2010, a total of 385,769 emergency department (ED) visits secondary to firearm-related injuries yielded 141,914 inpatient admissions with an estimated cost of more than 88 billion US dollars.^4^

Firearm injuries can result in a myriad of health outcomes, with both short- and long-term sequelae, including spinal cord injuries (SCI). Firearms are the main cause of traumatic spinal cord injuries in Brazil (28.4%). This rate varies from one country to another, dropping down to 8.4% in Thailand and as low as 1.9% in Turkey.^5^–^7^ In the US, 12.2% (784 out of a total of 17,730 new annual SCIs) are secondary to gunshot injuries.^8^,^9^ Spinal cord injuries also result in a significant health and financial burden at the level of the individual patient and their families, as well as at the level of the healthcare system. Less than 1% of affected individuals achieve complete neurological recovery upon hospital discharge, with the most frequent sequela being incomplete tetraplegia. Mortality rates are also highest during the first year post-injury.^10^

In contrast to most injuries that take priority in the management of trauma cases, SCIs can often be missed initially and not detected until later in the management process via imaging. They are often obscured by the presence and or need to manage more life-threatening concomitant injuries, particularly severe head trauma or hemorrhage, in addition to the performance of emergent procedures such as intubation, sedation, and surgical procedure.^6^,^11^–^12^

### Importance

This is the first study to identify general and clinical predictors of firearm-induced SCI and/or vertebral column injury (VCI), which would serve as cues for earlier detection and management of SCI/VCIs.

### Objectives

This study examines firearm-related spinal cord injuries in adult trauma patients in the US and identifies predictors of presence of such injuries in this patient population.

## METHODS

### Study Design and Setting

For this retrospective cohort study we used the public release dataset from the 2015 National Trauma Data Bank (NTDB). This dataset is an annually issued, US population-based, multicenter cohort and is considered the largest aggregation of US-based trauma registry data.^13^ The institutional review board at the American University of Beirut approved the use of the de-identified dataset to conduct this study.

### Selection of Participants

The total number of patients in the dataset was 917,865. The study sample included adult patients (≥16 years) who sustained firearm-induced injury coded under a list of *International Classification of Diseases, Ninth Revision* E codes “Mechanism” (Appendix) (N = 34,898). We excluded pediatric patients (age < 16 years, similar to other trauma studies^14^) and cases with missing age documentation (Figure).

**Population Health Research Capsule**

What do we already know about this issue?

*Firearm-related spinal cord injuries (SCI) are commonly missed in the initial assessment as they are often obscured by more life-threatening injuries*.

What was the research question?

*This study examines firearm-related SCI in adult trauma patients and identifies predictors of such injuries*.

What was the major finding of the study?

*SCI and/or vertebral column injury (VCI) were present in 7.9% of adult patients with trauma. Several clinical and non-clinical predictors were identified*.

How does this improve population health?

*The identified predictors can help with early detection of SCI/VCI injuries, avoid management delays, and improve outcomes of trauma patients*.

### Analysis

We conducted descriptive analyses to summarize the categorical variables by calculating their frequencies and percentages and to present the mean ± standard deviation (SD) of the continuous variables. Comparison of the percentages of all categorical variables according to the two groups of the cord injuries (none vs SCI and/or VCI) was done by using Pearson’s chi-square test. Due to the non-normal distribution, we used the Mann-Whitney test instead of Student’s t-test to compare the means of the continuous variables. More than 5% of the variables (ethnicity, whether patient used alcohol, whether patient used drug, the patient’s primary method of payment) were categorized as being not known/not recorded, and as a result we performed multiple imputation procedures to account for these missing data and thus to provide accurate estimates.

We conducted a multivariate logistic regression using a backward selection procedure to determine the predictors of SCI/VCI in patients with firearm-related injury. A receiving operating characteristic (ROC) curve was plotted to assess the validity of the logistic regression results. It indicated that the generated model discriminated excellently patients with no SCI or VCI from those with SCI and/or VCI (area under the ROC curve = 0.9, 95% confidence interval (CI), 0.88 – 0.89, *P*<0.001). Statistical significance was considered at an alpha value set at 0.05 and below. We performed analyses using the SPSS 24 (IBM Corporation, Armonk, NY) statistical package.

## RESULTS

### Characteristics of Study Subjects

#### Population and Hospital Characteristics (Table 1)

A total of 34,898 patients who sustained a firearm-induced injury were included in the analysis. Among those, 2768 patients (7.9%) had SCI or VCI. The mean age of patients with firearm-induced SCI and/or VCI was 30.1 (± 11.5 years), and 90.4% (N = 2501) were males.

### Main Results

#### Firearm Injury Characteristics and Locations (Table 2 and Table 3)

Firearm injuries associated with SCI and/or VCI were more likely to occur in public buildings, streets, and recreation areas (44.1% vs 38.5%; *P*-value <0.001). Assault (vs self-inflicted and unintentional injuries) was significantly higher in the SCI and/or VCI (86.4% vs 71.5%; *P*-value <0.001). Patients with SCI and/or VCI had more torso injuries (79.8% vs 44%; *P*-value <0.001); more head and neck injuries (28.1% vs 23.9%; *P*-value <0.001); and fewer injuries to the extremities (40.6% vs 60.1%; *P*-value <0.001); and fewer open wounds (52.5% vs 63.2%; *P*-value <0.001). Patients with SCI and/or VCI also commonly sustained more fractures (97.1% vs 53.0%; *P*-value <0.001), internal organ injuries (75.9% vs 35.6%; *P*-value <0.001), and blood vessel injuries (24.9% vs 12.2%; *P*-value <0.001). Patients with SCI and/or VCI more commonly had lower GCS score categories (severe [≤ 8] 22.7% vs 18.2%; *P*-value <0.001) and moderate [9–12] 4.5% vs 2.1%; *P*-value <0.001); lower systolic blood pressure (SBP≤ 90 millimeters mercury) (20.0% vs 12.7%; *P*-value <0.001); and higher Injury Severity Score (ISS) (≥ 16) (63.7% vs 26.0%; *P*-value <0.001).

#### Firearm Injury Outcomes (Table 3)

The mean length of hospital stay was significantly higher for patients with SCI and/or VCI (13.8 ± 17.3 days) compared to those with none (5.6 ± 9.4 days) (*P*-value <0.001). On the other hand, mortality rate in the ED or in hospital was not significantly different between the two groups (14.7%, N = 407 in SCI and/or VCI vs 15.0%, N = 4811 in no SCI or VCI group) (*P*-value = 0.703).

#### Predictors of SCI/VCI in Patients with Firearm-induced Injuries (Table 4)

##### General Predictors

After adjusting for important confounders, significant positive general predictors of presence of SCI and/or VCI included the following: assault injuries (odds ratio [OR] = 1.44; 95% CI, 1.17 – 1.79; Ref: Unintentional injuries); university hospital (OR = 1.16; 95% CI, 1.05 – 1.30; Ref: community hospital); public buildings, streets, or recreation sites as well as unspecified locations of injury (OR = 1.21; 95% CI, 1.07 – 1.36; Ref: home and residential institution); drug use (OR = 1.35; 95% CI, 1.22 – 1.49; Ref: No drug use); Medicaid coverage (OR = 1.19; 95% CI, 1.06 – 1.34; Ref: self-pay); and air medical transport (OR = 1.22; 95% CI, 1.06 – 1.41; Ref: ground ambulance). Increasing age was a slightly negative predictor for presence of SCI and/or VCI (OR = 0.995, 95% CI, 0.991 – 0.999).

##### Clinical Predictors

Additionally, the following positive clinical predictors were found to be significant for firearm-induced SCI and/or VCI: blood vessel injury (OR = 1.81; 95% CI, 1.60 – 2.05; Ref: no blood vessels injury); fractures (OR = 43.72; 95% CI, 33.94 – 56.32; Ref: no fractures); internal organ injury (OR = 1.38; 95% CI, 1.20 – 1.59; Ref: no internal organ injury); torso injury (OR = 3.25; 95% CI, 2.83 – 3.72; Ref: no torso injury); open wounds (OR = 1.19; 95% CI, 1.07 – 1.32; Ref: no open wounds); a mild or moderate GCS score (OR = 1.36; 95% CI, 1.19 – 1.55 and OR = 1.39; 95% CI, 1.06 – 1.81, respectively; Ref: severe GCS score [≤ 8]); and an ISS ≥ 16 (OR = 2.25; 95% CI, 2.00 – 2.53; Ref: ISS [<16]). Injury to extremities was a negative clinical predictor (OR = 0.32; 95% CI, 0.29 – 0.36; Ref: no extremity injury).

## DISCUSSION

This retrospective cohort study of 2768 patients who sustained a firearm-induced injury to the spinal cord or vertebral column is the largest to date to report on such injuries. With the exception of a study conducted by Jain et al on traumatic spinal cord injuries in general in the US,^8^ most studies were limited to small sample sizes and to single centers. Firearm-induced SCIs are relatively uncommon. The rate of SCI and/or VCI in firearm injuries in the current study was found to be 7.9%. This rate of SCI and/or VCI is lower than the previously reported rates of 10%^12^ and 23%^15^ among the civilian population, and the 11.10%^16^ rate of combat firearm injuries in the military population. The difference in rates across different studies is probably related to civilian vs military setting characteristics and firearms types.

While the mortality rate was not different among patients with SCI or VCI compared to those without, patients with firearm-induced SCI and/or VCI had more severe injuries than those without SCI or VCI. They more frequently had higher ISS, lower GCS scores, and lower SBP. These findings further reiterate the high impact of spinal injuries on affected victims in terms of clinical outcomes. However, this analysis may have missed patients with severe injuries or those who died from other major injuries, as they may not have survived long enough for evaluation for SCI and/or VCI.

Patients with SCI and/or VCI were more commonly found to have concomitant fractures, internal organ injuries, and blood vessel injuries compared to patients with no SCI or VCI. Furthermore, the injury location among patients with SCI and/or VCI involved the torso and head and neck more commonly than those with no SCI and/or VCI injury. These findings are in line with those of a previous study that examined patients who presented with gunshot wounds to the trunk, neck, or head over a 10-year period to a trauma center in Miami, Florida, where concomitant spine injuries were found in 10% of cases. It is worth noting that in the latter study, 13% of the detected cases of spine injuries were unsuspected, particularly when they involved the face (75%), abdomen (27%), chest (10%), shoulder (10%), back (5%), and flank (5%), but not the head.^12^

The mean length of hospital stay of 13.8 days (± 17.3) is slightly higher than the mean of 11 days reported by the National Spinal Cord Injury Statistical Center. Rehabilitation duration is not reported in the NTDB, but the national average rehabilitation length of stay is estimated to be around 31 days.^10^ The intensive care unit stay and ventilator days in the current study were also found to be significantly higher for patients with SCI and/or VCI compared to none. This translates into high healthcare costs secondary to firearm-induced SCI and/or VCI. According to the National Spinal Cord Injury Statistical Center, the average yearly expenses of affected individuals vary between US dollars $44,766 – $1,129,302, depending on the degree of neurological impairment, level of education, and pre-injury employment history.^10^ This is important in estimating the potential impact of the high cost of care of these injuries on patients and the government, especially given that a large portion of the study population is covered by Medicaid. Mitigation strategies, such as the adoption and enforcement of strict gun control laws, are needed to prevent such injuries and reduce their financial burden on affected victims.

This study is the first to identify predictors of firearm-induced SCI and/or VCI. A previous study examined prehospital predictors of traumatic spinal cord injuries in general: male gender; neurological deficit; altered mental status; high falls; diving injuries; and bike/motorbike collisions.^17^ Main predictors for firearm-induced SCI and/or VCI included unintentional injuries, assault forms of injuries, public or unspecified location of injuries, concomitant drug use by the subject, injury of the torso, as well as concomitant fractures, injuries to blood vessels, internal organs, or open wounds. Familiarity with these predictors is important for emergency providers, which would translate into earlier detection and management of SCI and/or VCI injuries and ultimately improved patient outcomes. Nevertheless, the full clinical utilization of such predictors, among others, would require further studies and the development and verification of clinical prediction rules.

## LIMITATIONS

This study did have a number of limitations. While the NTDB cohort is the largest registry representative of US-based trauma, some data elements that better characterize firearm-induced SCI and/or VCI (such as types of firearms, interval neurological examinations, and neurological outcomes at discharge) are not collected or reported. For instance, low GCS may be related to different factors and not limited to traumatic brain injury, which is not specified in the NTDB. While missing data is also considered a limitation of this study, the latter was addressed in the analysis via multiple imputations. Despite these limitations, the findings of this study, which used the NTDB dataset, apply in hospitals and trauma centers across the US and in similar clinical settings.

## CONCLUSION

Firearm-induced spinal cord and/or vertebral column injuries have a high burden on affected victims. This study identifies important general and clinical predictors for the presence of these injuries in trauma patients with firearm injuries. These predictors can help physicians suspect and detect the presence of SCI and/or VCI injuries for earlier management in order to improve outcomes of affected patients. Future studies involving databases with more detailed, neurological clinical data points can help further define the impact of such injuries on affected victims.

## Supplementary Information



## Figures and Tables

**Figure 1:**
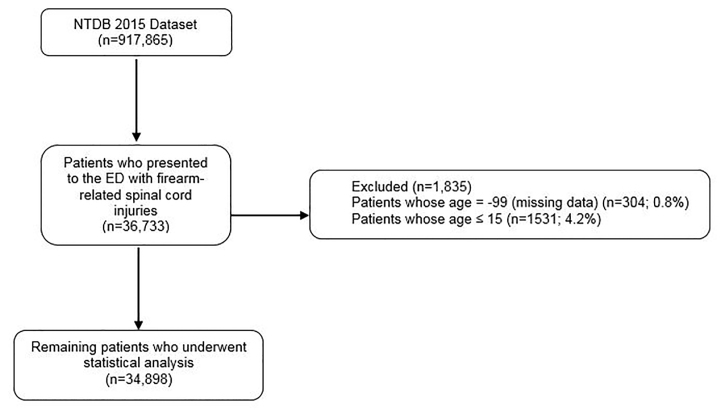
Flowchart showing inclusion and exclusion of patients with firearm-induced injuries. *NTDB*, National Trauma Data Bank ; *ED*, emergency department.

**Table 1:** Demographics of the general study population and the two groups: patients with no spinal cord injury (SCI) or vertebral column injury (VCI), and patients with SCI and/or VCI.

	General population (N = 34,898)	No SCI or VCI (N = 32,130)**	SCI and/or VCI (N = 2,768)	P-value‡
Age (years)	31.9 ± 13.5	32.1 ± 13.7	30.1 ± 11.5	<0.001*
Gender				
Female	3,867 (11.1%)	3,601 (11.2%)	266 (9.6%)	0.010
Male	31,022 (88.9%)	28,521 (88.8%)	2,501(90.4%)	
Not known/Not recorded	9 (0.0%)			
Race				
White	11,379 (32.6%)	10,704 (34.4%)	675 (25.2%)	<0.001
Black	18,686 (53.5%)	17,016 (54.6%)	1,670 (62.3%)	<0.001
Other race†	3,771(10.8%)	3,437 (11.0%)	334 (12.5%)	0.023
Not known/Not recorded	1,062 (3.0%)			
Hospital Teaching Status				
Community	11,127 (31.9%)	10,373 (32.3%)	754 (27.2 %)	<0.001
Non-teaching	3,327 (9.5%)	3,148 (9.8%)	179 (6.5 %)	<0.001
University	20,444 (58.6%)	18,609 (57.9%)	1,835 (66.3 %)	<0.001
State Designation				
Not applicable	3,039 (8.7%)	2,827 (8.8%)	212 (7.7%)	0.041
I	21,215 (60.8%)	19,334 (60.2%)	1,881 (68.0%)	<0.001
II	8,430 (24.2%)	7,857 (24.5%)	573 (20.7%)	<0.001
III	2,058 (5.9%)	1,965 (6.1%)	93 (3.4%)	<0.001
IV	65 (0.2%)	61 (0.2%)	4 (0.1%)	0.595
Other	91 (0.3%)	86 (0.3%)	5 (0.2%)	0.389
Hospital Geographic Region				
Northeast	4,537 (13.0%)	4,138 (13.0%)	399 (14.5%)	0.021
Midwest	6,837 (19.6%)	6,333 (19.8%)	504 (18.3%)	0.056
South	17,234 (49.4%)	15,877 (49.7%)	1,357 (49.3%)	0.700
West	6,095 (17.5%)	5,603 (17.5%)	492 (17.9%)	0.651
Missing	195 (0.6%)			
Patient’s Primary Method of Payment				
Self-Pay	11,927 (34.2%)	11,057 (34.4%)	870 (31.4%)	0.002
Medicaid	10,361 (29.7%)	9,352 (29.1%)	1,009 (36.5%)	<0.001
Medicare	1,822 (5.2%)	1,733 (5.4%)	89 (3.2%)	<0.001
Private/Commercial insurance	7,880 (22.6%)	7,304 (22.7%)	576 (20.8%)	0.020
Other Government	1,450 (4.2%)	1,353 (4.2%)	97 (3.5%)	0.074
Other and not billed (for any reason)	1,458 (4.2%)	1,331 (4.1%)	127 (4.6%)	0.261
Mode of Transportation				
Ground Ambulance	25,389 (72.8%)	23,288 (73.1%)	2,101 (76.2%)	<0.001
Air Medical Transport	3,864 (11.1%)	3,485 (10.9%)	379 (13.7%)	<0.001
Police	487 (1.4%)	436 (1.4%)	51 (1.8%)	0.040
Public/Private vehicle walk-in	4,474 (12.8%)	4,282 (13.4%)	192 (7.0%)	<0.001
Other	399 (1.1%)	365 (1.1%)	34 (1.2%)	0.680
Not known/not recorded	285 (0.8%)			

* The Mann-Whitney test was used to calculate the P-value.

** Missing values were disregarded when calculating percentages.

† “Other” race includes Asian, American Indian, Native Hawaiian, or other Pacific Islander and other race.

‡ P-values are comparing the “no SCI or VCI” group to the “SCI and/or VCI” group.

**Table 2:** Firearm injury characteristics and locations of the general study population and the two groups: patients with no spinal cord injury (SCI) or vertebral column injury (VCI) and patients with SCI and/or VCI.

	General population (N = 34,898)	No SCI or VCI (N =32,130)	SCI and/or VCI (N = 2,768)*	P-value
Injury intentionality as defined by the CDC Injury Intentionality Matrix				
Assault	25,348 (72.6%)	22,957 (71.5%)	2,391 (86.4%)	<0.001
Self-inflicted	3,766 (10.8%)	3,671 (11.4%)	95 (3.4%)	<0.001
Unintentional	4,050 (11.6%)	3,905 (12.2%)	145 (5.2%)	<0.001
Other and undetermined	1,734 (5.0%)	1,597 (5.0%)	137 (4.9%)	0.961
Location where injury occurred				
Home and residential institution	11,656 (33.4%)	10,936 (35.3%)	720 (27.1%)	<0.001
Industry, farm and mine	185 (0.5%)	171 (0.6%)	14 (0.5%)	0.870
Public building, street and recreation	13,116 (37.6%)	11,944 (38.5%)	1,172 (44.1%)	<0.001
Unspecified and other	8,691 (24.9%)	7,942 (25.6%)	749 (28.2%)	0.003
Not known/not recorded	1,250 (3.6%)			
Comorbidity				
No	16,728 (47.9%)	15,424 (48.0%)	1,304 (47.1 %)	0.036
Yes	18,170 (52.1%)	16,706 (52.0%)	1,464 (52.9 %)	
Alcohol use				
No	27,087 (77.6%)	24,978 (77.7%)	2,109 (76.2%)	0.061
Yes	7,811 (22.4%)	7,152 (22.3%)	659 (23.8%)	
Drug use				
No	25,710 (73.7%)	23,918 (74.4%)	1,792 (64.7%)	<0.001
Yes	9,188 (26.3%)	8,212 (25.6%)	976 (35.3%)	
Nature of injury as defined by the Barell Injury Diagnosis Matrix				
Blood vessels	4,597 (13.2%)	3,909 (12.2%)	688 (24.9%)	<0.001
Fractures	19,726 (56.5%)	17,037 (53.0%)	2,689 (97.1%)	<0.001
Internal organ	13,533 (38.8%)	11,432 (35.6%)	2,101 (75.9%)	<0.001
Open wounds	21,749 (62.3%)	20,297 (63.2%)	1,452 (52.5%)	<0.001
Others	3,902 (11.2%)	3,486 (10.8%)	416 (15.0%)	<0.001
Region 1: ICD-9 body region as defined by the Barell Injury Diagnosis Matrix				
Extremities	20,438 (58.6%)	19,315 (60.1%)	1,123 (40.6%)	<0.001
Head and neck	8,458 (24.2%)	7,681 (23.9%)	777 (28.1%)	<0.001
Spine and back	2,768 (7.9%)	0 (0%)	2,768 (100%)	<0.001
Torso	16,347 (46.8%)	14,138 (44.0%)	2,209 (79.8%)	<0.001
Unclassifiable by site	2,280 (6.5%)	2,016 (6.3%)	264 (9.5%)	<0.001
GCS Total (ED)				
Severe (≤ 8)	6,322 (18.1%)	5,708 (18.2%)	614 (22.7%)	<0.001
Moderate (9 – 12)	776 (2.2%)	655 (2.1%)	121 (4.5%)	<0.001
Mild (13 – 15)	26,994 (77.4%)	25,025 (79.7%)	1,969 (72.8%)	<0.001
Not known/not recorded	806 (2.3%)			
SBP (ED)				
≤ 90	4,520 (13.0%)	3,981 (12.7%)	539 (20.0%)	<0.001
≥ 91	29,427 (84.3%)	27,275 (87.3%)	2,152 (80.0%)	
Not known/not recorded	951 (2.7%)			
ISS				
< 16	24,245 (69.5%)	23,266 (74.0%)	979 (36.3%)	<0.001
≥ 16	9,877 (28.3%)	8,162 (26.0%)	1,715 (63.7%)	
Not Known/not recorded	776 (2.2%)			

* Missing values were disregarded when calculating percentages.

*ICD-9*, International Classification of Diseases, Ninth Edition; *CDC*, US Centers for Disease Control and Prevention; *GCS*, Glasgow Coma Scale Score; *ED*, emergency department; *SBP*, systolic blood pressure.

*SCI*, spinal cord injury*; VCI*, vertebral column injury; *ISS*, Injury Severity Score.

**Table 3:** Outcomes of the general study population and the two groups: patients with no spinal cord injury (SCI) or vertebral column injury (VCI) and patients with SCI and/or VCI.

	General population (N)	General population (Mean ± SD)	No SCI or VCI (N = 32,130)	SCI and/or VCI (N = 2,768)	P-value
Died in ED/hospital					
No	28,887 (82.8%)		26,608 (82.8%)	2,279 (82.3%)	0.521
Yes	5,218 (15.0%)		4,811 (15.0%)	407 (14.7%)	0.703
Not known/not recorded	793 (2.3%)				
Total length of stay in days	34,850	6.3 ± 10.45	5.6 ± 9.4	13.8 ± 17.3	<0.001*
Total number of days spent in the intensive care unit	11,883	6.0 ± 8.5	5.5 ± 7.7	9.0 ± 11.9	<0.001*
Total number of days spent on a ventilator	8,427	4.9 ± 7.8	4.3 ± 6.4	7.8 ± 12.7	<0.001*

* The Mann-Whitney test was used to calculate the P-values.

*SCI*, spinal cord injury; *VCI*, vertebral column injury; *ED*, emergency department; *SD*, standard deviation.

**Table 4:** Predictors of spinal cord injury/vertebral column injury in patients with firearm-induced injury.

	Odds ratio	95% CI	P-value
General predictors			
Age*	1	0.99–1.00	0.027
Hospital teaching status (community)			
Non-teaching	0.84	0.69 – 1.03	0.085
University	1.16	1.05 – 1.30	0.006
Injury Intentionality as defined by the CDC Injury Intentionality Matrix (Unintentional)			
Self-inflicted	0.31	0.23 – 0.42	<0.001
Assault	1.44	1.17 – 1.79	0.001
Other and undetermined	1.25	0.93 – 1.68	0.144
Location where injury occurred (Home & residential institution)			
Industry, farm and mine	1.15	0.58 – 2.27	0.698
Public building, street and recreation	1.21	1.07 – 1.36	0.002
Unspecified and other	1.2	1.05 – 1.37	0.008
Drug use			
Yes	1.35	1.22 – 1.49	<0.001
The patient’s primary method of payment (self-pay)			
Medicaid	1.19	1.06 – 1.34	0.004
Medicare	1.07	0.81 – 1.42	0.628
Private/commercial insurance	1.06	0.92 – 1.21	0.412
Other government	0.82	0.62 – 1.07	0.146
Other and not billed (for any reason)	1.1	0.87 – 1.39	0.441
Mode of transportation (Ground Ambulance)			
Air Medical Transport	1.22	1.06 – 1.41	0.007
Police	0.61	0.41 – 0.90	0.013
Public/private vehicle walk-in	0.71	0.59 – 0.86	<0.001
Other	0.94	0.62 – 1.44	0.782
Clinical predictors			
Nature of injury as defined by the Barell Injury Diagnosis Matrix (Reference: No)			
Blood vessel injury	1.81	1.60 – 2.05	<0.001
Fractures	43.72	33.94 – 56.32	<0.001
Internal organ injury	1.38	1.20 – 1.59	<0.001
Open wounds	1.19	1.07 – 1.32	0.001
Extremities injury	0.32	0.29 – 0.36	<0.001
Torso injury	3.25	2.83 – 3.72	<0.001
GCS total (ED) (Severe (≤ 8))			
Moderate (9 – 12)	1.39	1.06 – 1.81	0.016
Mild (13 – 15)	1.36	1.19 – 1.55	<0.001
ISS (≤ 15)			
≥ 16	2.25	2.00 – 2.53	<0.001

* Rounded up: 3-decimal odds ratio for age = 0.995; 95% confidence interval [0.991 – 0.999.]

*CI*, confidence interval; GCS, Glasgow Coma Scale Score; *ED*, emergency department; *SBP*, systolic blood pressure; *ISS*, Injury Severity Score.
