# Supplementary material for: Patterns and Predictors of Firearm-related Spinal Cord Injuries in Adult Trauma Patients
Source: West J Emerg Med. 2021 Feb 15;22(2):270–7. doi: 10.5811/westjem.2020.9.48202 (PMC7972377; doi:10.5811/westjem.2020.9.48202)
Supplement: Supplementary file 1 [file wjem-22-270-s001.docx]

**Appendix**

Inclusion Criteria for ICD-9 Injury Mechanism E-Codes

| Name | Label | Category/ Numbers Selected | | | | | |
| --- | --- | --- | --- | --- | --- | --- | --- |
| MECHANISM | ICD-9-CM Mechanism of Injury E-Code | Firearm | | | | | |
| ECODE | ICD-9-CM External Cause of Injury Code | '922.0' | '922.8' | '955.4' | '965.3' | '985.1' | '990.2' |
|  |  | '922.1' | '922.9' | '955.6' | '965.4' | '985.2' | '990.3' |
|  |  | '922.2' | '955.0' | '955.7' | '968.6' | '985.3' | '990.9' |
|  |  | '922.3' | '955.1' | '965.0' | '970' | '985.4' | '991.0' |
|  |  | '922.4' | '955.2' | '965.1' | '979.4' | '985.6' | '991.1' |
|  |  | '922.5' | '955.3' | '965.2' | '985.0' | '985.7' | '991.2' |
